# The acquisition of *Clostridium tyrobutyricum* mutants with improved bioproduction under acidic conditions after two rounds of heavy-ion beam irradiation

**DOI:** 10.1038/srep29968

**Published:** 2016-07-18

**Authors:** Xiang Zhou, Zhen Yang, Ting-Ting Jiang, Shu-Yang Wang, Jian-Ping Liang, Xi-Hong Lu, Liang Wang

**Affiliations:** 1 Institute of Modern Physics, Chinese Academy of Sciences,509 Nanchang Rd., Lanzhou, Gansu 730000, P. R. China; 2 Nanjing Agricultural University, Nanjing 210095, P. R. China; 3 University of Chinese Academy of Sciences, Beijing 100049, P. R. China

## Abstract

End-product inhibition is a key factor limiting the production of organic acid during fermentation. Two rounds of heavy-ion beam irradiation may be an inexpensive, indispensable and reliable approach to increase the production of butyric acid during industrial fermentation processes. However, studies of the application of heavy ion radiation for butyric acid fermentation engineering are lacking. In this study, a second ^12^C^6+^ heavy-ion irradiation-response curve is used to describe the effect of exposure to a given dose of heavy ions on mutant strains of *Clostridium tyrobutyricum*. Versatile statistical elements are introduced to characterize the mechanism and factors contributing to improved butyric acid production and enhanced acid tolerance in adapted mutant strains harvested from the fermentations. We characterized the physiological properties of the strains over a large pH value gradient, which revealed that the mutant strains obtained after a second round of radiation exposure were most resistant to harsh external pH values and were better able to tolerate external pH values between 4.5 and 5.0. A customized second round of heavy-ion beam irradiation may be invaluable in process engineering.

*Clostridium tyrobutyricum* (*C. tyrobutyricum*) is a gram-positive, rod-shaped, spore-forming obligate anaerobic bacterium^1^,^2^. Its main fermentation products are butyric and acetic acid from various carbohydrates, including glucose, xylose, fructose, disaccharides, sucrose and lactose^3^,^4^,^5^. The generation of butyric acid from natural resources by *C. tyrobutyricum* plays a key role in many scientific and industrial processes^6^,^7^. Environmental concerns have revived interest in the use of renewable resources and microbial fermentation technologies for the production of valuable fuels and chemicals^8^. *C. tyrobutyricum* also releases hydrogen, which has a high energy content per unit weight (141.86 kJ/g or 61,000 Btu/lb) and is considered one of the most promising replacements for fossil fuels^8^,^9^,^10^. The metabolic pathway and key enzymes involved in butyrate and acetate production in *C. tyrobutyricum* have been studied extensively from genetic and metabolic engineering perspectives^6^,^12^. Recent studies have reported a novel strain capable of producing large amounts of n-butanol called ACKKO-adhE2, which was developed by introducing a butanol synthesis pathway into the high butyrate-producing mutant ACKKO in conjunction with a downregulated acetate formation pathway via advanced metabolic engineering and synthetic biology technologies^12^,^13^. The production of butyrate by biofermentation enables greater fermentative feedstock diversity, and fibrous-bed bioreactors featuring cell immobilization systems and cell recycling have been designed to increase reactor productivity, butyrate yield, and final product concentrations^14^,^15^. However, final product inhibition is one of the key factors limiting organic acid production by biofermentation; the production of acetate as a byproduct of metabolic processes not only decreases butyrate yield but also leads to increased costs associated with product recovery and purification. In the last years, the development of recombinant DNA technology and other related technologies has provided new tools for approaching yields improvement by means of genetic manipulation of biosynthetic pathway. However, improved strains have been generated by heavy-ion beam irradiation and selection for the development of commercial strains for use in microbial fermentation processes^16^,^17^,^18^,^19^. ^12^C^6+^ heavy-ion irradiation may represent an inexpensive and reliable approach for increasing the productivity of industrial processes. Extensive efforts have focused on improving the butyric acid titres of *C. tyrobutyricum* by ^12^C^6+^ heavy-ion irradiation^16^,^20^. We observed that carbon ions effectively induced the expression of key enzymes in glycolysis, product formation, energy and redox metabolism, and the cellular response to butyric acid production. It is crucial to distinguish mutants that are acid-tolerant, and well-adapted mutant strains were characterized for their physiological properties, including their ability to survive ^12^C^6+^ heavy-ion irradiation, expression status during the fermentation process, cell growth, changes in response to pH variation, butyric acid tolerance, butyrate/acetate ratio, and gas production. Evaluating these characteristics often involves a quantitative approach to the acquisition and analysis of data, and an efficient discovery process is required to extract information from the data. In this study, we investigated the utility of two rounds of heavy-ion beam irradiation to create *C. tyrobutyricum* mutants with improved bioproduction capability under acidic conditions. Furthermore, the introduction of versatile statistical methods enabled a thorough characterization of the productivity and acid tolerance of the secondary mutant.

## Results and Discussion

### Lethality of two doses of ^12^C^6+^ heavy-ion irradiation

In this study, a ^12^C^6+^ heavy-ion irradiation-response curve was generated after a second round of radiation and was used to describe the effects of a specific dose of ^12^C^6+^ heavy ions on the *C. tyrobutyricum* strain (No. H51-8-4). Table 1 shows the sample points that were randomly selected before any data-processing tasks were performed; this selection was not biased by a human operator. *x* denotes the irradiation dose of ^12^C^6+^ heavy ions. As shown in Table 1, lethality increases with increasing *x*. These experiments consisted of 16 independent irradiation tests. The mortality rate increases with increasing irradiation dose. In Table 1, at the lowest irradiation dose of 15 Gy, *n* = 50 × 10^2^ mutant cells. For each cell, the outcome is either death or survival. *y* denotes the number of mutant strain cells that die and is thus a random variable with abinomial distribution. The probability that *y* assumes a value *k*, where *k* = 0; 1…, 50 × 10^2^, thus has a binomial distribution in which the success probability *p* depends on the irradiation dose *x* and is given by the following formula:




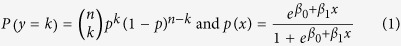




where *β*_*0*_ and *β*_*1*_ are the regression parameters and *p(x)* varies between 0 and 1. We then transform the formula to a logistic regression function:




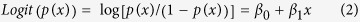




In the random ^12^C^6+^ heavy-ion irradiation samples, the lowest irradiation dose was *x*_*1*_ = 15 Gy; *n*_*1*_ = 50 × 10^2^ mutant cells were irradiated at this dose, resulting in *y*_*1*_ = 5 × 10^2 ^cell deaths. The likelihood function is the following:




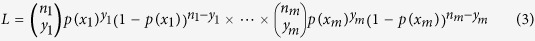




where *L* = *β*_*0*_ and *β*_*1*_ are as in function (3). The logistic regression parameters are as follows:




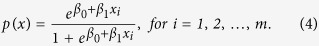




**Figure 1:**
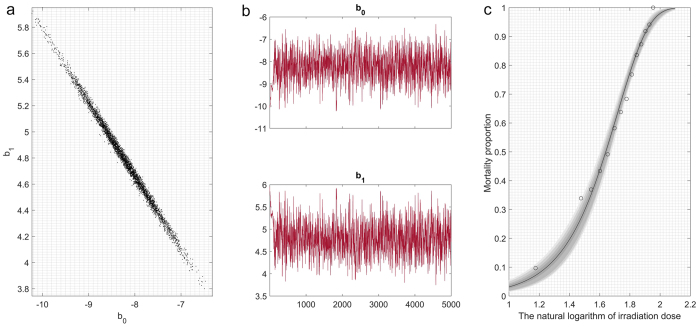
Modelling lethality as a function of the irradiation dose via Markov Monte Carlo chain, delayed rejection and adaptive metropolis. (**a**) The left panel shows the 1D parameter chains. (**b**) The middle panel shows the 2D Markov Monte Carlo chain using 5.0 × 10^3^ consecutive steps of delayed rejection and adaptive metropolis. (**c**) The right panel indicates that the model fits a randomly selected subset of the chain, and the model’s predictive envelope was calculated. The grey areas of the plot correspond to the 50%, 90%, 95%, and 99% posterior regions (see the web version of the article).

We calculated predictive *p* values by comparing the model deviance diagnostics [−2 × log(likelihood function)] calculated by comparing the experimental radiation value observations from the posterior predictive distribution over the Markov chain Monte Carlo. The values were used to calculate the model fit for a randomly selected subset of the chain as well as the predictive envelope of the model. The 2D Markov Monte Carlo chain (Fig. 1a) and the 1D parameter chain (Fig. 1b) lengths were fixed at 5000 steps for all dimensions to achieve reliable random experimental data. For each dimension, the runs were repeated 500 times. The data yielded an estimated logistic regression model relating the radiation mortality of the mutant *C. tyrobutyricum* strain (No. H51-8-4) to ^12^C^6+^ heavy ions at an energy input of 240 AMeV and a dose of log (15–90 Gy) and the estimated logistic regression curve (Fig. 1c). The grey areas correspond to 50%, 90%, 95%, and 99% posterior regions. The *p* value in this study was the probability of observing a larger deviance following experimental radiation than the actual data indicate. No such discrepancy was observed. In this work, we describe in detail a previously unknown lethality trend following a second round of ^12^C^6+^ heavy-ion irradiation of *C. tyrobutyricum.* Complex, slow death curves have often been constructed in an attempt to explain a set of experimental data; however, these curves were not sufficiently accurate to discriminate between different irradiation doses. In the classical regression framework, the present study seeks to model a continuous response variable y as a function of one or more predictor variables. Most regression problems are of this type. In the radiation experiment, the measured outcome of interest is either a survival or death, which we can code as a 1 or a 0. The probability of a survival or death may depend on a set of predictor variables. This type of data could be modelled by simply fitting a regression with the goal of estimating the probability of success given some values of the predictor. However, this approach will not work because probabilities are constrained to fall between 0 and 1^21^,^22^. In the classical regression setup with a continuous response, the predicted values can range over all real numbers. Therefore, a different modelling technique is needed.

### Evaluation of mutant strain expression states by principal component analysis

**Figure 2:**
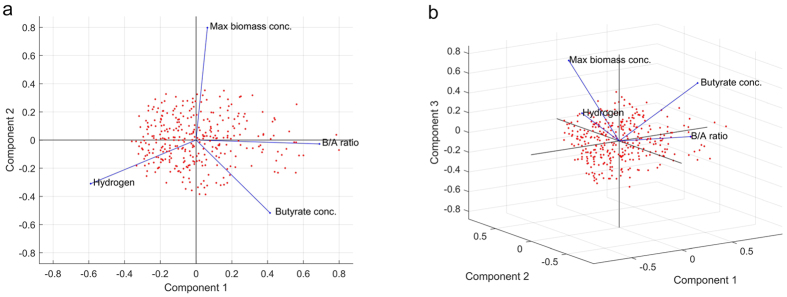
Analysis of mutant physiological properties during culture in serum bottles using weighted principal component analysis. **(a)** The left panel is a visualization of the orthonormal principal component coefficients for each variable (maximum biomass concentration, butyrate concentration, hydrogen and B/A ratio) and the principal component scores of each observation in a single plot. The data are plotted based on the first two principal components with approximately 62% confidence (θ = 0.62). **(b)** The right panel is a 3D plot including a point for each of the 329 strains. The coordinates indicate the score of each strain for the three principal components in the graph. Using the first three principal components, approximately 93% confidence (θ = 0.93) was achieved, indicating that the error between the original dataset and the projected dataset was less than 7%.

In general, the producing ability of mutant strains is limited by survival rates of 10.2–11.7% and is reflected by an increased *Y*_*butyric acid*_:*Y*_*acetic acid*_ ratio (B/A ratio)^23^,^24^. We randomly extracted and mixed selected mutants from the irradiation sample (80–85 Gy), including No. W87-M-7, No. P327-45-9 and No. G271-81-36. These mixed samples were named No. FS-ZKJ-D_80–85_ and classified into fifteen groups, each containing ten samples. Based on the experimental steps in the method, 329 mutants were screened. The data include ratings for 3 different indicators of mutation quality among the 329 screened strains exposed to ^12^C^6+^ heavy ions at an energy input of 240 AMeV and a dose of 80–85 Gy. The results for these mutants confirm previous results obtained for *C. tyrobutyricum* grown in chemically defined Reinforced *Clostridial* SEM-defined P2 medium (in serum bottles) containing a glucose-limited chemostat culture after ^12^C^6+^ heavy-ion irradiation. These conditions yielded the maximum biomass concentration, butyrate concentration, hydrogen, and B/A ratio during the first 54 hours of fermentation. A higher rating corresponds to a superior irradiated mutant strain. Figure 2a presents a principal component analysis scatter plot generated for the 329 screened strains. The correlation among some variables was as high as 0.93, and independent new variables that are linear combinations of the original variables were obtained. As shown in Table S1, because the data are 4-dimensional, the covariance matrix will be 4 × 3. We will therefore only provide the three principal component coefficient vectors:




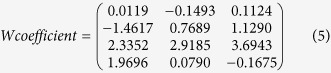




These coefficients are weighted; hence the coefficient matrix is not orthonormal. Transforming the coefficients so that they are orthonormal yields the following:




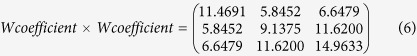




All three variables are represented in this bi-plot by a vector, and the direction and length of the vector indicate how each variable contributes to the two principal components in Fig. 2a. The first principal component has positive coefficients with three variables (maximum biomass concentration, butyrate concentration and B/A ratio) and a negative coefficient with hydrogen. However, the second principal component has positive coefficients with all variables. When all variables are in the same unit, it is appropriate to compute principal components for raw data. When variables have different units or the difference in the variance of different columns is substantial, principal component analysis can be performed using the inverse variances of the ratings as weights. The results indicated that No. FS-ZKJ-D_80–85_-8, No. FS-ZKJ-D_80–85_-78, No. FS-ZKJ-D_80–85_-150, No. FS-ZKJ-D_80–85_-203, No. FS-ZKJ-D_80–85_-221, No. FS-ZKJ-D_80–85_-278 and No. FS-ZKJ-D_80–85_-323 are outliers compared to the remainder of the 329 screened strains. Then, a statistical measure of the multivariate distance of each observation from the centre of the data set was performed using Hotelling’s *T*^*2*^, which indicated that No. FS-ZKJ-D_80–85_-8 was the most extreme among the strains. Figure 2b presents a 3-D (three dimensions) plot that includes a point for each of the 329 strains irradiated with ^12^C^6+^ heavy ions with an energy input of 240 AMeV and a dose 80–85 Gy. The coordinates indicate the score of each determining data point for the two principal components in the figure. Points near the left edge of this plot had the lowest scores for the first principal component. The points are scaled with respect to the maximum score value and maximum coefficient length, and thus only their relative locations can be determined from the graph. The first principal component explains a sufficient proportion of the variance in our determining data. The series of experiments of ^12^C^6+^ heavy-ion irradiation and screening of mutant *C. tyrobutyricum* strains produced observations of differential expression for hundreds of mutant strains across multiple conditions. The application of principal component analysis to the expression data in this study allowed a core set of independent features of the expression states of the 329 mutant strains to be compared directly. Our analysis explains the differences in the mutant strains’ expression states and can be used to simplify the analysis and visualization of multidimensional data sets.

### Investigating the specific effect of externally added butyrate on mutant cell growth

**Figure 3:**
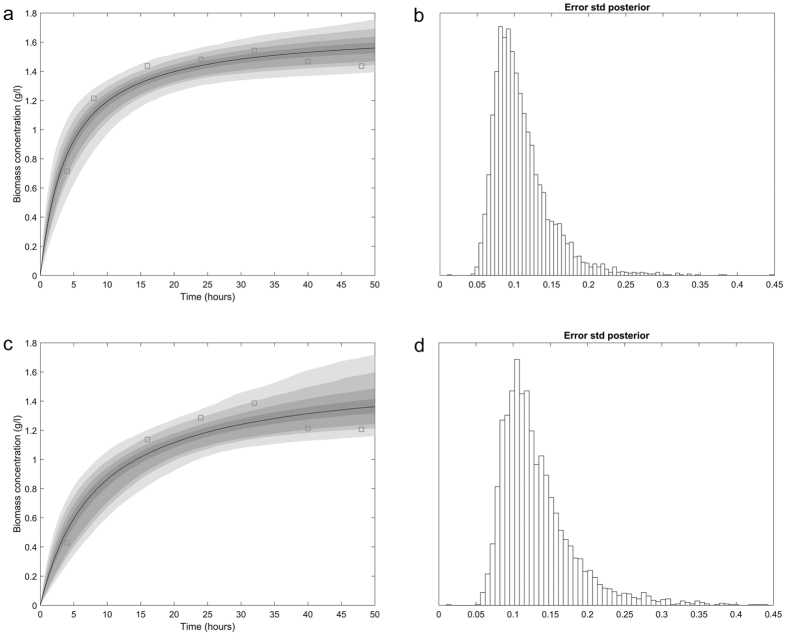
The predictive posterior distribution of the effect of the butyrate concentration gradient on the experimental biomass concentration of the mutant cells. (**a**) Growth in medium containing 50 g/L glucose and supplemented with 10.0 g/L butyric acid. The model fit for a randomly selected subset of the chain and calculation of the predictive envelope of the model are shown. The grey areas in the plot correspond to 50%, 90%, 95%, and 99% posterior regions (see the web version of the article). (**b**) A histogram of the chain of the square root of the s2chain for error (i.e., standard deviation). (**c**) Growth in medium containing 50 g/L glucose and supplemented with 12.0 g/L butyric acid. The model fit for a randomly selected subset of the chain and calculation of the predictive envelope of the model are shown. The grey areas in the plot correspond to 50%, 90%, 95%, and 99% posterior regions (see the web version of the article). (**d**) A histogram of the chain of the square root of the s2chain for error (i.e., standard deviation).

Previous studies have demonstrated that regardless of the strain used, cell growth is gradually inhibited, with no notable growth at butyric acid concentrations of greater than 8 g/L. We assessed the effect of added butyrate on the fermentation of the mutants obtained by two rounds of heavy-ion beam irradiation. The cell-growth profiles of No. FS-ZKJ-D_80–85_-8 were compared as shown in Fig. 3 during the first 50 hours of a classical growth trend. Individual batch cultures were conducted in chemically defined P2-medium (in serum bottles) containing 50 g/L glucose and supplemented with 10.0 g/L butyric acid. Using *x*_*m*_ = 1.543 g/L dry weight and *x* = 0.830 g/L dry weight from our experimental data, a plot of equation (10) yielded a slope *μ*_*m*_ of 0.1934 1/h and an intercept of (−0.0732). The calculated value of *x*_*0*_, 0.816 g/L dry weight, is lower than the experimental value, possible due to differences in the number of viable cells during the culture process. Mutant cell viability of less than 100% may yield an *x*_*0*_ value smaller than the measured initial mutant cell concentration. In Fig. 3a, the top left panel shows the biomass concentration, *x*, which was calculated based on equations (8 and 9) using the values of *x*_*0*_ and *μ*_*m*_ as determined above, and the value of *x*_*m*_, which was also obtained from the experimental data, and compares the predicted and observed values of the biomass concentration. This model has a tendency to overestimate the biomass concentration when cell growth enters the stationary phase after 32 h of fermentation and underestimate the biomass concentration in the exponential phase. This result is coincident with the underestimated value of *x*_*0*._ The chain variable is a nsimu × npar matrix. The square root of the s2chain yields the chain for the error standard deviation, as depicted in Fig. 3b in the top right panel. Individual batch cultures were conducted in chemically defined P2-medium (in serum bottles) containing 50 g/L glucose and supplemented with 12.0 g/L butyric acid. Taking *x*_*m*_ = 1.386 g/L dry weight and *x* = 0.612 g/L dry weight from our experimental data, a plot of equation (10) yielded a slope *μ*_*m*_ of 0.1434 1/h and an intercept of (−0.0547). The calculated value of *x*_*0*_, 0.553 g/L dry weight, was lower than that of the experimental value. This discrepancy might be attributable to deviations in the number of viable cells during the culture process. Mutant cell viability of less than 100% may yield an *x*_0_ value less than the measured initial mutant cell concentration. As shown in Fig. 3c, the lower left panel shows the biomass concentration, *x*, which was calculated based on equations (8 and 9) using the values of *x*_*0*_ and *μ*_*m*_ as determined above, and the value of *x*_*m*_, which was also obtained from the experimental data, and compares the predicted and observed values of the biomass concentration. This model has a tendency to overestimate the biomass concentration when the cell growth enters the stationary phase after 32 h of fermentation and underestimate biomass concentration in the exponential phase. This result is coincident with the underestimated value of *x*_*0*._ The chain variable is a nsimu × npar matrix, and the square root of the s2chain yields the chain for the error standard deviation, as depicted in Fig. 3d in the lower right panel. As fully expected, after a short lag phase, No. FS-ZKJ-D_80–85_-8 exhibited a biphasic metabolic pattern strongly influenced by the the two different pH values of the medium. The mutant cells entered the exponential growth phase coincident with the initiation of the production of butyric acid. Our studies demonstrate that No. FS-ZKJ-D_80–85_-8 did not exhibit gradual inhibition of cell growth, with notable growth at butyric acid concentrations of greater than 12 g/L. In addition, as a general trend, at high pH, organic acids are mainly formed, whereas at low pH, solvent production is stimulated. However, these differences in pH regulate the temporal switch associated with solvent formation by the mutant strain, which exhibits its own intrinsic genetic and metabolic characteristics. Butyric acid strongly inhibited cell growth of the wild-type strain, whereas No. FS-ZKJ-D_80–85_-8 was less strongly inhibited. The growth inhibition by butyric acid can be partially attributed to the inhibition effect on key enzymes in the metabolic pathway of *C. tyrobutyricum*^25^,^26^,^27^. The enzymes in the phosphotransacetylase → acetate kinase pathway in *C. tyrobutyricum* are more sensitive to butyric acid inhibition than those in the phosphotransbutyrylase → butyrate kinase pathway^1^,^2^,^10^,^11^. Due to the disruption of the *ack* gene by radiation and partial impairment of the phosphotransacetylase → acetate kinase pathway, No. FS-ZKJ-D_80–85_-8 generates ATP mainly from the phosphotransbutyrylase → butyrate kinase pathway and is less sensitive to butyric acid inhibition. Therefore, No. FS-ZKJ-D_80–85_-8 requires more time to adapt and respond to higher butyrate concentrations when primarily relying on the phosphotransacetylase → acetate kinase pathway for energy supply. These obligate fermentative mutants have adapted to the consequences of their lifestyle. It is not the purpose of this study to provide an extensive explanation based on these inherent physiological properties but to assign possible causes for the effects observed, supported by available studies documented in the literature.

### pH variation in serum bottles

**Figure 4:**
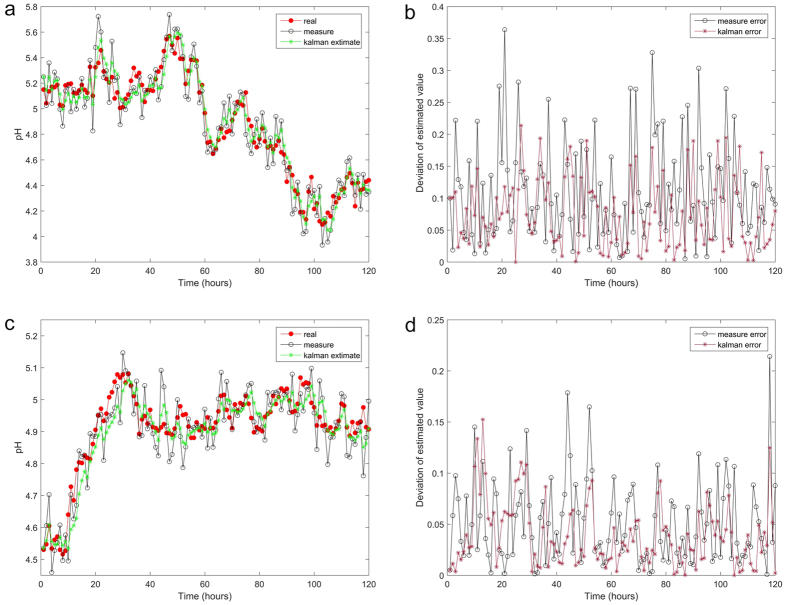
Trends in pH value variation assessed using vector algebra for minimum mean squared estimator-compiled statistical analysis using the Kalman filter algorithm. (**a**) Kalman filter analysis of mutants maintained in serum bottles at an initial pH of 5.0. (**b**) The measurement error and Kalman error for linear systems. This result suggests a one-dimensional linear system with measurement errors drawn from a zero-mean Gaussian distribution. (**c**) Kalman filter analysis of mutants maintained in serum bottles at an initial pH of 4.5. (**d**) The measurement error and Kalman error for linear systems. This result suggests a one-dimensional linear system with measurement errors drawn from a zero-mean Gaussian distribution.

We determined how fluctuations in the pH of the growth medium around a set point affected the metabolic properties of No. FS-ZKJ-D_80–85_-8. In general, an external pH value below 5.0 (optimal pH value 4.5) and an endogenous pH greater than 5.5 are required to induce solventogenesis. In this study, the pH of the serum bottles was adjusted to pH 5.0 or pH 4.5 using a sodium butyrate acid buffer solution when necessary. The pH value was measured, and recorded every two hours. All measurement data were analysed using vector algebra and minimum mean squared estimator-compiled statistical analysis according to the Kalman filter algorithm^28^,^29^,^30^. The Kalman filter is an algorithm that permits exact inference in a linear dynamic system (see the Wikipedia entry for the Kalman filter). The measured error and Kalman error are indicated in Fig. 4b,d for both linear systems. This result indicates that for a one-dimensional linear system with measurement errors drawn from a zero-mean Gaussian distribution, our two models yield the optimal estimator. As shown in Fig. 4a,c, No. FS-ZKJ-D_80–85_-8 exhibited pH drops to approximately 4.2 (ΔpH of 0.8 starting from 5.0) and 4.9 (ΔpH of 0.4 starting from 4.5) during the first 120 hours of growth in the serum bottles. Interestingly, the highest pH value was observed at 42 hours when the initial pH was 5.0 (Fig. 4a) and at 28 hours when the initial pH was 4.5 (Fig. 4c). This phenomenon is attributable to the synthesis of neutral solvent from a pre-existing acidic product to detoxify the environment^31^,^32^,^33^. It is a reasonable generalization that No. FS-ZKJ-D_80–85_-8 produces acidic fermentation products when growing at lower pH. Two examples of this phenomenon that have been well studied are the production of butyrate from a larger pH gradient by No. FS-ZKJ-D_80–85_-8. As shown in Fig. 4c, in the latter instance, the induction of the new metabolic pathway is not the result of the perturbation of the pH value. Rather, in both pH value variations, the basis of this phenomenon appears to be the accumulation of acidic fermentation products in the medium, resulting in a decline in the pH value, and accumulation of acid in the cytoplasm due to the resulting transmembrane pH gradient. High intracellular concentrations of acid induce enzymes that produce neutral solvent products. Thus, No. FS-ZKJ-D_80–85_-8 synthesizes the enzymes necessary for butyrate production at lower pH if the pH gradient in the medium is increased. Comparing the pH trend in the serum bottles, as shown in Fig. 4a,c, revealed that the role of the external pH is to create a larger pH gradient to ensure that induction occurs before the external concentration of acid becomes sufficiently high to inhibit growth of No. FS-ZKJ-D_80–85_-8. Furthermore, a comparative analysis of both linear systems clearly reveals one major cluster composed by No. FS-ZKJ-D_80–85_-8 with similar overall tolerance to an increasing pH gradient when compared with the two initial pH values. As shown in Fig. 4a,c, the two drops in pH value demonstrate that perturbing the external pH value has a prominent inhibitory effect on mutant cell growth, with all specific growth rates declining with increasing initial pH value. This finding confirms that the *C. tyrobutyricum* No. FS-ZKJ-D_80–85_-8 is the most resistant to a critical external pH value and demonstrate greater “apparent” tolerance to the real external pH value region of 4.5–5.0. Thus, the improved pH gradient tolerance allowed No. FS-ZKJ-D_80–85_-8 to produce more butyric acid at a higher final concentration, as demonstrated by the fermentation quantitative assessment study discussed below.

### Quantitative assessment of the influence of external pH range on bioproduction

**Figure 5:**
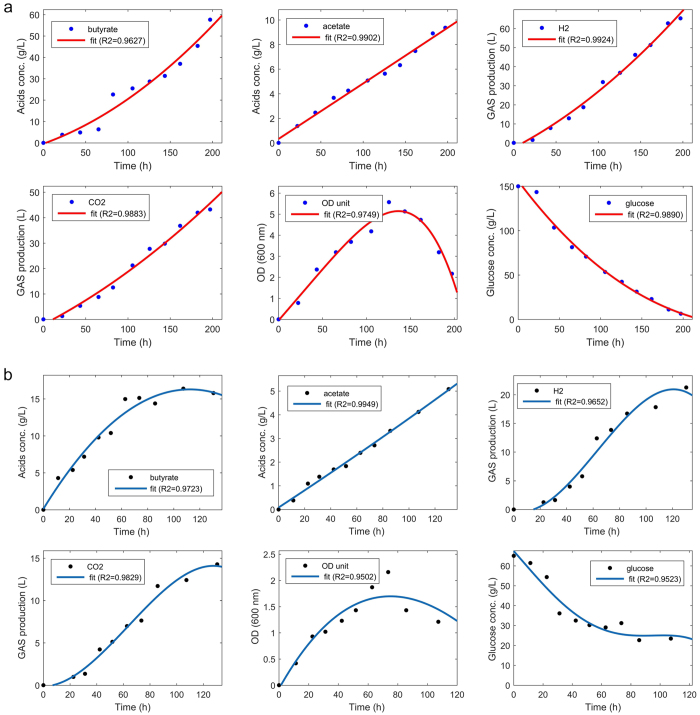
Quantitative assessment of the influence of external pH value range on bioproduction via fermentation kinetics. **(a)** Butyric acid, acetic acid, GAS production, change in OD600, and glucose consumption at different time points during biofermentation by the mutant strains in media containing concentration gradients of 150 g/L glucose at 37 °C and an initial pH of 5.0 over 200 h of biofermentation. **(b)** Butyric acid, acetic acid, GAS production, change in OD600, and glucose consumption at different time points during biofermentation by mutant strains in media containing concentration gradients of 60 g/L glucose at 37 °C and an initial pH of 4.5 over 110 h of biofermentation.

Figure 5 presents the kinetics of glucose fermentations at pH 5.0 (Fig. 5a) and pH 4.5 (Fig. 5b) at 37 °C by No. FS-ZKJ-D_80–85_-8. In general, mutant cells grew exponentially during the first 30–40 hours, then entered the stationary phase. The cells continued to produce butyrate until metabolism terminated entirely at a higher butyrate gradient that inhibited the mutant cells. According to the literature, wild-type fermentation produces butyrate at a much lower final concentration (21.34–19.98 g/L at pH 6.0 and 37 °C). However, as shown in Fig. 5a (pH 5.0 and 37 °C), the mutant strain produced butyrate at a much higher final concentration (approximately 57.63 g/L) than the wild-type strains. The higher butyrate concentration produced by the mutant is consistent with the higher tolerance of the mutant to butyric acid inhibition. Figure 5b summarizes the quantitative assessment of fermentation by No. FS-ZKJ-D_80–85_-8 at pH 4.5 and 37 °C with glucose as the substrate. The kinetics and yield of fermentation differed greatly for the mutant cells grown on glucose at pH 5.0 and pH 4.5, respectively. Compared with the two different initial pH values used during fermentation, glucose metabolism is less energy efficient and consequently resulted in a lower specific growth rate and biomass yield, as shown in Fig. 5b. Interestingly, the mutants grown using glucose as the substrate shifted their metabolism away from major butyric acid production at an initial pH of 5.0 to primarily yield acetic acid at an initial pH of 4.5. However, in these mutants, the disruption of the *ack* gene by radiation did not significantly affect acetate formation using glucose as the fermentation substrate at two different initial pH values. As shown in Fig. 5, acetic acid production from glucose did not differ significantly between pH 5.0 and pH 4.5 after 100 hours. However, more butyrate was produced by the mutant, and the butyrate/acetate ratio (B/A) increased from 6.2:1 at pH 5.0 to 2.9:1 at pH 4.5. Thus, the additional round of heavy-ion beam irradiation disrupted the *ack* gene in these mutants, resulting in increased carbon flux toward the phosphotransbutyrylase → butyrate kinase pathway. However, a larger pH gradient at an initial pH of 4.5, as shown in Fig. 5b, revealed that the phosphotransbutyrylase → butyrate kinase pathway is greatly impaired in the mutant. Furthermore, at the initial pH of 5.0, which is close to the *pKa* value butyric acid of 4.89, most of the butyric acid was present in the form of free acid, which is relatively easily recovered by solvent extraction (Fig. 5a)^34^,^35^. The microbial metabolism of butyrate as a sole acid product in the fermentation industry incentivizes reducing its separation and purification costs. In addition, as shown in Fig. 5a, the production of hydrogen during metabolism was also increased in the mutant. This result was attributed to the enhanced hydrogenase activity of enzymes following the second round of ^12^C^6+^ heavy-ion irradiation. Although this result was not anticipated based on the original heavy-ion irradiation experimental design, this is the first time that hydrogen production by a *C. tyrobutyricum* mutant has been improved by irradiation from an engineering perspective: more hydrogen was produced by the mutant, perhaps because the mutant needed to maintain the redox balance by converting more NAD^+^ to NADH to compensate for the reduction in energy efficiency due to reduced flux through the phosphotransbutyrylase → butyrate kinase pathway^34^,^36^,^37^. In summary, a second round of ^12^C^6+^ heavy-ion irradiation affected global metabolic pathways in *C. tyrobutyricum* and metabolic flux changes through various metabolites, including butyric acid, acetic acid, carbon dioxide and hydrogen, due to the need for redox balance and the redistribution of carbon and energy. Further studies are needed to fully understand the underlying causes of the mutant’s improved production of butyrate.

## Methods

### Experimental setup and heavy-ion beam irradiation

Heavy-ion beam experimental setups were employed as previously described^16^,^18^,^19^,^20^. Briefly, the extraction time for ^12^C^6+^ heavy ions with 240 AMeV of energy was approximately 3 s, and the priming dose was 15–90 Gy. The dose rates were as high as 10 Gy/min. In this study, the operating parameters were as follows: the radiation energy input was 240 AMeV, the distance between the ^12^C^6+^ heavy ion nozzle exit and the spore suspension was 3.5 mm, 200 and 100 cells/well of the mutant strains (No. H51-8-4) were plated to accommodate the different sizes of the wells in 6-well plates and 96 well-plates (6,000 and 3,000 cells/well for No. H51-8-4) and were allowed to attach overnight. ^12^C^6+^ heavy-ion irradiation was performed on the following day. The temperature of the irradiation treatment was maintained at <38 °C under these conditions in a vacuum.

### Comparison of survival by MTT assay

The survival fraction was determined as previously described^16^,^38^. Briefly, Dulbecco’s modified Eagle’s medium (DMEM, Gibco Glasgow, UK) was supplemented with 100 μL of MTT reagent (c = 0.5 g/L) in each well and incubated for 30 min at 37 °C. The MTT assay was performed in 96-well plates containing 5,000–6,500 cells per well and analysed using the following equation^16^,^38^.




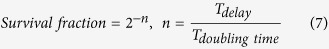




where *T*_*delay*_ = the time period to reach a specific absorption value for control versus irradiated cells, and *T*_*doubling time*_ = the time period required for a quantity of cells to double.

### Growth and culture medium

The mutant *C. tyrobutyricum* strains ATCC 25755 and No. H51-8-4 were cultured anaerobically at 37 °C in a previously described synthetic clostridial growth medium. The optimal culture medium consisted of the following: 3.6 g/L yeast extract, 3.2 g/L corn steep flour, 2.7 g/L peptone, 3.2 g/L K_2_HPO_4_, 1.24 g/L NaHCO_3_, 3.2 g/L KH_2_PO_4_, 0.2 g/L MgSO_4_, 0.2 g/L MnSO_4_, 0.02 g/L FeSO_4_, 0.02 g/L CaCl, (Difco, Detroit, MI, USA), 2.5 g/L ammonium acetate, 0.0006 g/L *p*-aminobenzoate, 0.0006 g/L thiamin, 0.00006 g/L biotin and 37 μg/ml thiamphenicol. Culture was performed in an anaerobic chamber.

### Assessment of microbial growth using the logistic equation

In this study, based on our previous experimental data obtained for multiple biofermentation systems such as polysaccharide fermentation by the mutant strain, cell growth was characterized by the following logistic formula^39^,^40^.




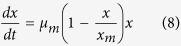




where *μ*_*m*_ = the maximum growth rate (1/h) and *x*_*m*_ = the maximum attainable biomass concentration. When *x = x*_*0*_ (t = 0), the logistic formula yields a sigmoid variation of *x* as a formula of t, which represents both an exponential and a stationary phase^41^,^42^.




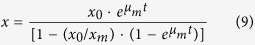







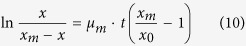




where *x*_*m*_ = the experimental data. A linear regression of the linearized data yields a line of slope *μ*_*m*_ and intercept *y*.

### Experiments in serum bottles

Medium supplemented with different concentrations of butyrate of 10–12 g/L were added to the serum bottles and adjusted to pH 4.5 and pH 5.0 using a sodium butyrate/butyric acid buffer solution. Each serum bottle was purged with nitrogen for 15 min to attain total anaerobic conditions and was sealed, autoclaved at 123 °C, 15 psig for 25 min, and maintained at room temperature. Dextrose solution was subsequently added to obtain a final glucose concentration of 35 g/L, followed by inoculation with 3.5 mL of fresh mutant strain culture. The final volume of the cell suspension in medium was 150 mL. The serum bottles containing different butyrate concentrations were incubated at 37 °C for different fermentation periods, and samples were collected periodically to measure pH, optical density, residual glucose concentration, and acid and solvent production.

### Bioreactor fermentation

The inoculum was prepared as previously described, and anaerobic fermentations were performed in a 7–L BioFlo^®^/CelliGen^TM^ 115 bioreactor/fermentor (New Brunswick Scientific Co., Edison, NJ) containing 2 L of reinforced *Clostridial* SEM P2 medium with 250 mL of inoculum. The initial glucose concentration in the medium varied from 0 to 180 g/L. The temperature, pH and agitation speed were maintained at 37 °C, pH 4.5–5.0 and 150 rpm, respectively, for the duration of the culture period in stirred-tank bioreactors, guaranteeing a sufficient oxygen-free nitrogen supply at a flow rate of 0.4–0.45 L/min. In this study, the use of a concentrated solution for fermentation was also investigated. The optimal concentrations were as follows: 500 g/L glucose, 25 g/L MgSO_4_·7H_2_O, 1.3 g/L MnSO_4_ and 0.6 g/L FeSO_4_.

### Analytical methods

A high-performance liquid chromatography (HPLC) system was used to analyse carbohydrate compounds, including glucose, in the fermentation broth. The HPLC system consisted of an automatic injector (Agilent 1100, G1313A), a pump (Agilent 1100, G1311A), a Zorbax carbohydrate analysis column (250 mm × 4.6 mm, 5 μm; Agilent, USA), a column oven maintained at 30 °C (Agilent 1100, G1316A), and a refractive index detector (Agilent 1100, G1362A). The mobile phase was ethyl nitrile (ethyl nitrile/water = 75:25) at a flow rate of 1.5 mL/min. Butyric acid and acetic acid were analysed with a GC-2014 Shimadzu gas chromatograph (GC) (Shimadzu, Columbia, MD, USA) equipped with a flame ionization detector and a 30.0-m fused silica column (0.25-mm film thickness and 0.25-mm ID, Stabilwax-DA). The GC was operated at an injection temperature of 200 °C, and a 1-μL sample was injected using an AOC-20i Shimadzu autoinjector. The column temperature was maintained at 80 °C for 3 min, increased to 150 °C at a rate of 30 °C/min, and maintained at 150 °C for 3.7 min. The cell density was analysed by measuring the optical density (OD) of the cell suspension at 600 nm using a spectrophotometer (Thermo Spectronics, Genesys 20, USA) with a conversion of 0.396 ± 0.012 g/L of dry cell weight (DCW) per OD unit. The elemental carbon, hydrogen, oxygen, and nitrogen contents were measured with a Sercon-GSL (CEInstruments, Milan, Italy). Gas hydrogen and carbon dioxide production in the biofermentation mixture were monitored using an online respirometer system equipped with both hydrogen and carbon dioxide sensors (Micro-oxymax system, Columbus Instrument, Columbus, OH).

### Statistical analysis

Predicted simulation values were analysed using the terms of the Markov chain Monte Carlo method^21^,^22^,^43^, principal component analysis (PCA), the Kalman filter algorithm^28^,^29^,^30^, the sum-of-squares function, the Jacobian matrix of the vector function, logistic regression, loglog counting and probit regression.

## Additional Information

**How to cite this article**: Zhou, X. *et al*. The acquisition of *Clostridium tyrobutyricum* mutants with improved bioproduction under acidic conditions after two rounds of heavy-ion beam irradiation. *Sci. Rep.* **6**, 29968; doi: 10.1038/srep29968 (2016).

## Supplementary Material

Supplementary Information

## Figures and Tables

**Table 1:** Random sampling points after ^12^C^6+^ heavy-ion irradiation with an energy input of 240 AMeV and a dose of 15 ~ 90 Gy.

Sample No. H51-8-4	Irradiation dose	Log of irradiation dose	Total of cells strains	Total of cells strains lethal
No. 1	15 Gy	1.1761	50 × 10^2^	600
No. 2	20 Gy	1.3010	62 × 10^2^	850
No. 3	25 Gy	1.3979	55 × 10^2^	800
No. 4	30 Gy	1.4771	59 × 10^2^	2000
No. 5	35 Gy	1.5441	65 × 10^2^	2400
No. 6	40 Gy	1.6021	60 × 10^2^	2600
No. 7	45 Gy	1.6532	57 × 10^2^	2800
No. 8	50 Gy	1.6989	55 × 10^2^	3200
No. 9	55 Gy	1.7403	58 × 10^2^	3700
No. 10	60 Gy	1.7782	60 × 10^2^	4100
No. 11	65 Gy	1.8129	56 × 10^2^	4300
No. 12	70 Gy	1.8451	67 × 10^2^	5600
No. 13	75 Gy	1.8575	63 × 10^2^	5500
No. 14	80 Gy	1.9031	61 × 10^2^	5600
No. 15	85 Gy	1.9294	68 × 10^2^	6400
No. 16	90 Gy	1.95424	70 × 10^2^	7000
