# Supplementary material for: The acquisition of Clostridium tyrobutyricum mutants with improved bioproduction under acidic conditions after two rounds of heavy-ion beam irradiation
Source: Sci Rep. 2016 Jul 18;6:29968. doi: 10.1038/srep29968 (PMC4947956; doi:10.1038/srep29968)
Supplement: Supplementary Information [file srep29968-s1.pdf]

Supplementary Information

The acquisition of *Clostridium tyrobutyricum* mutants with improved bioproduction under acidic conditions after two rounds of heavy-ion beam irradiation

Xiang Zhou [1, *], Zhen Yang[1, 2], Ting-Ting Jiang [1, 3], Shu-Yang Wang[1], Jian-Ping Liang[1], Xi-Hong Lu[1] & Liang Wang[1, 3]

Author affiliations

1 Institute of Modern Physics, Chinese Academy of Sciences, 509 Nanchang Rd., Lanzhou, Gansu 730000, PR China

2 Nanjing Agricultural University, Nanjing 210095, PR China

3 University of Chinese Academy of Sciences, Beijing 100049, PR China

***Corresponding author** E-mail: syannovich@gmail.com

*Corresponding author

Xiang Zhou, Phone: 86-931-4969688 Fax: +86-931-8272100

E-mail: syanovich@gmail.com

Supplementary Information: 12 pages, 1 table

Table S1 329 mutant's strain expression states, individual batch cultures were carried out in chemically defined P2-medium (performed in serum bottles) containing 35 g/L glucose at 37°C, pH=6.0~6.5 over 62 h of biofermentation.

No. mutants	*Maximum biomass concentration (g/L)*	*Hydrogen production (L)*	*Butyric acid concentration (g/L)*	*$Y_{butyric\ acid}$:$Y_{acetic\ acid}$ ratio (B/A) ratio*
FS-ZKJ-D_{80-85}-1	1.417	13.466	5.666	0.758907
FS-ZKJ-D_{80-85}-2	1.452	10.302	21.976	5.108322
FS-ZKJ-D_{80-85}-3	1.175	7.771	7.6	4.291361
FS-ZKJ-D_{80-85}-4	1.512	13.264	14.254	1.96228
FS-ZKJ-D_{80-85}-5	1.599	12.288	17.187	2.733302
FS-ZKJ-D_{80-85}-6	1.492	14.113	12.549	1.546777
FS-ZKJ-D_{80-85}-7	1.735	12.937	6.109	0.88064
FS-ZKJ-D_{80-85}-8	1.567	7.249	22.049	17.65332
FS-ZKJ-D_{80-85}-9	1.493	8.872	22.617	7.875
FS-ZKJ-D_{80-85}-10	1.647	7.556	8.883	5.708869
FS-ZKJ-D_{80-85}-11	1.539	10.483	18.1	4.037475
FS-ZKJ-D_{80-85}-12	1.2	8.324	22.807	9.813683
FS-ZKJ-D_{80-85}-13	1.468	8.218	23.881	10.76691
FS-ZKJ-D_{80-85}-14	1.15	11.284	23.704	4.485995
FS-ZKJ-D_{80-85}-15	1.566	13.604	10.5	1.380852
FS-ZKJ-D_{80-85}-16	1.462	10.651	7.986	1.71705
FS-ZKJ-D_{80-85}-17	1.13	15.751	12.45	1.276792
FS-ZKJ-D_{80-85}-18	1.228	10.475	11.677	2.609385
FS-ZKJ-D_{80-85}-19	1.122	15.29	7.589	0.8169
FS-ZKJ-D_{80-85}-20	1.262	11.725	16.852	2.943581
FS-ZKJ-D_{80-85}-21	1.681	11.4	9.131	1.690926
FS-ZKJ-D_{80-85}-22	1.706	10.567	6.739	1.475586
FS-ZKJ-D_{80-85}-23	1.588	12.538	6.202	0.948608
FS-ZKJ-D_{80-85}-24	1.24	15.387	11.878	1.265367
FS-ZKJ-D_{80-85}-25	1.393	9.848	17.002	4.418399
FS-ZKJ-D_{80-85}-26	1.553	14.739	8.812	1.008353

FS-ZKJ-$D_{80\text{-}85}$-27	1.553	14.195	6.693	0.816718
FS-ZKJ-$D_{80\text{-}85}$-28	1.636	10.84	17.796	3.67686
FS-ZKJ-$D_{80\text{-}85}$-29	1.364	15.226	15.506	1.680685
FS-ZKJ-$D_{80\text{-}85}$-30	1.647	12.675	8.208	1.229663
FS-ZKJ-$D_{80\text{-}85}$-31	1.474	11.355	11.236	2.098226
FS-ZKJ-$D_{80\text{-}85}$-32	1.7	9.539	8.599	2.429782
FS-ZKJ-$D_{80\text{-}85}$-33	1.751	7.413	11.627	8.228592
FS-ZKJ-$D_{80\text{-}85}$-34	1.693	13.584	22.344	2.946203
FS-ZKJ-$D_{80\text{-}85}$-35	1.17	14.344	7.689	0.9215
FS-ZKJ-$D_{80\text{-}85}$-36	1.254	13.325	13.453	1.836587
FS-ZKJ-$D_{80\text{-}85}$-37	1.21	11.405	15.939	2.948936
FS-ZKJ-$D_{80\text{-}85}$-38	1.776	13.321	9.149	1.249693
FS-ZKJ-$D_{80\text{-}85}$-39	1.54	7.798	11.947	6.644605
FS-ZKJ-$D_{80\text{-}85}$-40	1.319	10.834	13.376	2.767067
FS-ZKJ-$D_{80\text{-}85}$-41	1.463	11.578	10.779	1.932413
FS-ZKJ-$D_{80\text{-}85}$-42	1.593	14.919	21.37	2.396009
FS-ZKJ-$D_{80\text{-}85}$-43	1.324	13.74	11.326	1.463307
FS-ZKJ-$D_{80\text{-}85}$-44	1.574	12.425	16.579	2.580389
FS-ZKJ-$D_{80\text{-}85}$-45	1.428	14.379	13.439	1.603891
FS-ZKJ-$D_{80\text{-}85}$-46	1.503	12.722	14.313	2.129277
FS-ZKJ-$D_{80\text{-}85}$-47	1.375	11.706	9.93	1.740273
FS-ZKJ-$D_{80\text{-}85}$-48	1.127	13.895	21.073	2.669158
FS-ZKJ-$D_{80\text{-}85}$-49	1.272	15.139	9.909	1.084254
FS-ZKJ-$D_{80\text{-}85}$-50	1.501	7.43	10.803	7.554545
FS-ZKJ-$D_{80\text{-}85}$-51	1.734	14.418	9.021	1.071632
FS-ZKJ-$D_{80\text{-}85}$-52	1.47	13.783	12.745	1.637543
FS-ZKJ-$D_{80\text{-}85}$-53	1.657	9.658	7.64	2.088573
FS-ZKJ-$D_{80\text{-}85}$-54	1.314	11.778	13.919	2.408965
FS-ZKJ-$D_{80\text{-}85}$-55	1.604	13.268	9.258	1.273803
FS-ZKJ-$D_{80\text{-}85}$-56	1.363	12.407	17.37	2.711097
FS-ZKJ-$D_{80\text{-}85}$-57	1.259	15.03	12.664	1.402436
FS-ZKJ-$D_{80\text{-}85}$-58	1.674	13.06	7.585	1.074363
FS-ZKJ-$D_{80\text{-}85}$-59	1.589	10.851	18.101	3.731396

FS-ZKJ-D_{80-85}-60	1.754	11.593	6.219	1.111926
FS-ZKJ-D_{80-85}-61	1.163	11.316	8.723	1.640895
FS-ZKJ-D_{80-85}-62	1.476	15.178	15.171	1.652975
FS-ZKJ-D_{80-85}-63	1.431	14.972	17.652	1.967454
FS-ZKJ-D_{80-85}-64	1.196	13.255	20.002	2.756995
FS-ZKJ-D_{80-85}-65	1.212	14.592	13.478	1.568669
FS-ZKJ-D_{80-85}-66	1.689	12.183	13.415	2.169659
FS-ZKJ-D_{80-85}-67	1.742	10.911	12.777	2.60171
FS-ZKJ-D_{80-85}-68	1.616	11.39	8.329	1.545269
FS-ZKJ-D_{80-85}-69	1.58	11.732	7.42	1.294487
FS-ZKJ-D_{80-85}-70	1.31	12.638	6.996	1.053932
FS-ZKJ-D_{80-85}-71	1.167	15.542	11.966	1.254035
FS-ZKJ-D_{80-85}-72	1.531	15.104	11.931	1.310523
FS-ZKJ-D_{80-85}-73	1.634	12.22	15.51	2.493569
FS-ZKJ-D_{80-85}-74	1.662	11.75	13.629	2.370261
FS-ZKJ-D_{80-85}-75	1.508	13.618	14.943	1.961538
FS-ZKJ-D_{80-85}-76	1.655	11.9	23.052	3.907119
FS-ZKJ-D_{80-85}-77	1.579	12.29	16.789	2.669157
FS-ZKJ-D_{80-85}-78	1.151	7.686	21.555	12.7847
FS-ZKJ-D_{80-85}-79	1.707	10.199	21.551	5.132412
FS-ZKJ-D_{80-85}-80	1.418	13.495	13.542	1.806805
FS-ZKJ-D_{80-85}-81	1.67	14.826	15.376	1.742126
FS-ZKJ-D_{80-85}-82	1.53	14.439	17.8	2.109255
FS-ZKJ-D_{80-85}-83	1.607	10.115	21.736	5.282139
FS-ZKJ-D_{80-85}-84	1.186	12.993	24.358	3.483197
FS-ZKJ-D_{80-85}-85	1.605	14.897	5.853	0.657862
FS-ZKJ-D_{80-85}-86	1.407	14.471	22.612	2.669342
FS-ZKJ-D_{80-85}-87	1.636	15.212	22.457	2.437799
FS-ZKJ-D_{80-85}-88	1.221	13.538	6.953	0.922393
FS-ZKJ-D_{80-85}-89	1.128	14.594	8.72	1.014661
FS-ZKJ-D_{80-85}-90	1.712	8.37	13.301	5.612236
FS-ZKJ-D_{80-85}-91	1.647	7.614	14.744	9.135068
FS-ZKJ-D_{80-85}-92	1.227	10.273	18.688	4.373508

FS-ZKJ-D_{80-85}-93	1.295	14.688	24.483	2.818025
FS-ZKJ-D_{80-85}-94	1.552	8.127	24.503	11.51998
FS-ZKJ-D_{80-85}-95	1.258	7.79	16.528	9.23352
FS-ZKJ-D_{80-85}-96	1.403	15.533	22.759	2.387391
FS-ZKJ-D_{80-85}-97	1.384	11.588	7.807	1.397101
FS-ZKJ-D_{80-85}-98	1.724	8.738	14.313	5.227538
FS-ZKJ-D_{80-85}-99	1.546	9.378	7.722	2.285968
FS-ZKJ-D_{80-85}-100	1.186	10.131	21.769	5.269668
FS-ZKJ-D_{80-85}-101	1.592	15.245	10.206	1.103948
FS-ZKJ-D_{80-85}-102	1.756	14.639	12.797	1.481306
FS-ZKJ-D_{80-85}-103	1.511	8.309	16.204	7.017757
FS-ZKJ-D_{80-85}-104	1.52	15.242	10.242	1.108202
FS-ZKJ-D_{80-85}-105	1.422	14.78	24.266	2.763781
FS-ZKJ-D_{80-85}-106	1.242	10.941	5.63	1.139445
FS-ZKJ-D_{80-85}-107	1.68	12.543	22.134	3.382852
FS-ZKJ-D_{80-85}-108	1.72	10.223	10.472	2.479754
FS-ZKJ-D_{80-85}-109	1.547	8.173	13.24	6.092959
FS-ZKJ-D_{80-85}-110	1.361	13.686	12.515	1.628285
FS-ZKJ-D_{80-85}-111	1.648	15.484	10.333	1.089519
FS-ZKJ-D_{80-85}-112	1.169	15.273	18.188	1.961393
FS-ZKJ-D_{80-85}-113	1.153	11.348	14.137	2.643418
FS-ZKJ-D_{80-85}-114	1.415	15.762	21.865	2.239807
FS-ZKJ-D_{80-85}-115	1.491	10.537	7.248	1.597531
FS-ZKJ-D_{80-85}-116	1.664	10.754	13.354	2.809003
FS-ZKJ-D_{80-85}-117	1.518	12.75	18.217	2.698815
FS-ZKJ-D_{80-85}-118	1.681	9.219	9.798	3.043802
FS-ZKJ-D_{80-85}-119	1.543	10.699	12.929	2.751436
FS-ZKJ-D_{80-85}-120	1.216	9.076	12.967	4.21554
FS-ZKJ-D_{80-85}-121	1.6	11.869	16.222	2.764014
FS-ZKJ-D_{80-85}-122	1.137	13.524	24.608	3.270601
FS-ZKJ-D_{80-85}-123	1.677	8.825	12.881	4.559646
FS-ZKJ-D_{80-85}-124	1.352	15.564	5.785	0.604872
FS-ZKJ-D_{80-85}-125	1.214	11.961	24.607	4.127999

FS-ZKJ-$D_{80\text{-}85}$-126	1.28	13.891	13.509	1.71195
FS-ZKJ-$D_{80\text{-}85}$-127	1.689	8.96	19.571	6.611824
FS-ZKJ-$D_{80\text{-}85}$-128	1.733	12.345	7.729	1.218125
FS-ZKJ-$D_{80\text{-}85}$-129	1.552	8.973	16.991	5.715103
FS-ZKJ-$D_{80\text{-}85}$-130	1.377	15.811	9.008	0.918153
FS-ZKJ-$D_{80\text{-}85}$-131	1.205	9.348	19.727	5.892174
FS-ZKJ-$D_{80\text{-}85}$-132	1.36	9.419	11.728	3.430243
FS-ZKJ-$D_{80\text{-}85}$-133	1.198	8.587	13.866	5.359876
FS-ZKJ-$D_{80\text{-}85}$-134	1.578	7.374	14.754	10.73799
FS-ZKJ-$D_{80\text{-}85}$-135	1.256	11.32	10.67	2.005639
FS-ZKJ-$D_{80\text{-}85}$-136	1.462	8.563	24.63	9.609832
FS-ZKJ-$D_{80\text{-}85}$-137	1.659	13.483	10.137	1.354671
FS-ZKJ-$D_{80\text{-}85}$-138	1.274	8.418	8.413	3.479322
FS-ZKJ-$D_{80\text{-}85}$-139	1.391	12.646	20.896	3.144147
FS-ZKJ-$D_{80\text{-}85}$-140	1.254	12.476	15.72	2.427424
FS-ZKJ-$D_{80\text{-}85}$-141	1.57	11.04	23.348	4.63254
FS-ZKJ-$D_{80\text{-}85}$-142	1.328	12.042	21.062	3.485932
FS-ZKJ-$D_{80\text{-}85}$-143	1.674	15.583	15.605	1.628404
FS-ZKJ-$D_{80\text{-}85}$-144	1.236	12.846	23.549	3.439819
FS-ZKJ-$D_{80\text{-}85}$-145	1.225	13.241	23.972	3.310592
FS-ZKJ-$D_{80\text{-}85}$-146	1.551	15.326	16.562	1.775895
FS-ZKJ-$D_{80\text{-}85}$-147	1.534	10.838	14.901	3.079992
FS-ZKJ-$D_{80\text{-}85}$-148	1.231	13.982	5.797	0.726259
FS-ZKJ-$D_{80\text{-}85}$-149	1.396	8.855	18.203	6.375832
FS-ZKJ-$D_{80\text{-}85}$-150	1.447	7.593	20.253	12.71375
FS-ZKJ-$D_{80\text{-}85}$-151	1.267	12.12	14.674	2.397712
FS-ZKJ-$D_{80\text{-}85}$-152	1.309	10.273	9.486	2.219986
FS-ZKJ-$D_{80\text{-}85}$-153	1.763	10.406	11.679	2.650704
FS-ZKJ-$D_{80\text{-}85}$-154	1.374	12.53	18.53	2.837672
FS-ZKJ-$D_{80\text{-}85}$-155	1.569	15.693	8.519	0.878882
FS-ZKJ-$D_{80\text{-}85}$-156	1.497	10.444	16.708	3.759676
FS-ZKJ-$D_{80\text{-}85}$-157	1.299	14.407	13.858	1.648388
FS-ZKJ-$D_{80\text{-}85}$-158	1.466	8.003	19.248	9.609586

FS-ZKJ-D_{80-85}-159	1.493	11.09	16.77	3.294695
FS-ZKJ-D_{80-85}-160	1.5	12.055	6.577	1.08621
FS-ZKJ-D_{80-85}-161	1.293	10.044	13.976	3.455984
FS-ZKJ-D_{80-85}-162	1.148	9.696	11.42	3.089827
FS-ZKJ-D_{80-85}-163	1.498	8.345	19.066	8.13049
FS-ZKJ-D_{80-85}-164	1.573	12.177	23.105	3.740489
FS-ZKJ-D_{80-85}-165	1.429	8.898	21.158	7.300897
FS-ZKJ-D_{80-85}-166	1.386	13.742	19.455	2.512917
FS-ZKJ-D_{80-85}-167	1.374	8.638	24.956	9.460197
FS-ZKJ-D_{80-85}-168	1.778	9.445	10.65	3.091437
FS-ZKJ-D_{80-85}-169	1.425	11.563	6.447	1.158907
FS-ZKJ-D_{80-85}-170	1.773	14.869	8.589	0.968429
FS-ZKJ-D_{80-85}-171	1.527	9.66	21.154	5.779781
FS-ZKJ-D_{80-85}-172	1.179	13.159	8.794	1.228384
FS-ZKJ-D_{80-85}-173	1.525	13.941	7.999	1.007304
FS-ZKJ-D_{80-85}-174	1.709	13.211	14.574	2.021079
FS-ZKJ-D_{80-85}-175	1.627	10.753	23.364	4.915632
FS-ZKJ-D_{80-85}-176	1.361	14.963	7.53	0.84012
FS-ZKJ-D_{80-85}-177	1.42	13.014	8.931	1.273311
FS-ZKJ-D_{80-85}-178	1.583	9.254	10.024	3.080516
FS-ZKJ-D_{80-85}-179	1.775	11.597	22.055	3.940504
FS-ZKJ-D_{80-85}-180	1.338	15.142	23.444	2.564428
FS-ZKJ-D_{80-85}-181	1.721	13.879	8.024	1.018403
FS-ZKJ-D_{80-85}-182	1.418	10.768	16.389	3.43729
FS-ZKJ-D_{80-85}-183	1.675	8.303	24.688	10.71993
FS-ZKJ-D_{80-85}-184	1.592	7.796	13.045	7.263363
FS-ZKJ-D_{80-85}-185	1.334	13.242	12.878	1.778238
FS-ZKJ-D_{80-85}-186	1.714	9.05	10.469	3.432459
FS-ZKJ-D_{80-85}-187	1.283	9.193	10.129	3.172252
FS-ZKJ-D_{80-85}-188	1.428	7.524	10.774	7.069554
FS-ZKJ-D_{80-85}-189	1.178	13.217	6.582	0.912013
FS-ZKJ-D_{80-85}-190	1.474	9.474	22.541	6.488486
FS-ZKJ-D_{80-85}-191	1.504	10.509	6.31	1.399423

FS-ZKJ-D_{80-85}-192	1.734	9.255	16.229	4.985868
FS-ZKJ-D_{80-85}-193	1.628	11.081	22.47	4.422358
FS-ZKJ-D_{80-85}-194	1.523	15.714	18.413	1.895512
FS-ZKJ-D_{80-85}-195	1.151	11.052	23.823	4.715558
FS-ZKJ-D_{80-85}-196	1.586	8.754	11.228	4.076979
FS-ZKJ-D_{80-85}-197	1.554	14.35	15.064	1.804072
FS-ZKJ-D_{80-85}-198	1.399	9.899	7.212	1.849705
FS-ZKJ-D_{80-85}-199	1.698	10.523	24.857	5.495689
FS-ZKJ-D_{80-85}-200	1.432	9.77	6.324	1.677454
FS-ZKJ-D_{80-85}-201	1.138	12.065	22.315	3.679308
FS-ZKJ-D_{80-85}-202	1.431	10.6	18.976	4.125217
FS-ZKJ-D_{80-85}-203	1.775	7.293	16.888	13.0611
FS-ZKJ-D_{80-85}-204	1.305	15.407	23.974	2.548528
FS-ZKJ-D_{80-85}-205	1.19	14.284	24.199	2.921173
FS-ZKJ-D_{80-85}-206	1.701	11.087	10.116	1.988598
FS-ZKJ-D_{80-85}-207	1.386	10.218	18.016	4.271219
FS-ZKJ-D_{80-85}-208	1.176	10.646	22.053	4.746664
FS-ZKJ-D_{80-85}-209	1.318	11.807	11.535	1.986396
FS-ZKJ-D_{80-85}-210	1.14	13.944	11.501	1.447759
FS-ZKJ-D_{80-85}-211	1.259	13.998	18.353	2.294699
FS-ZKJ-D_{80-85}-212	1.459	8.774	21.792	7.855804
FS-ZKJ-D_{80-85}-213	1.489	7.888	9.34	4.947034
FS-ZKJ-D_{80-85}-214	1.769	7.946	9.797	5.03443
FS-ZKJ-D_{80-85}-215	1.325	12.254	20.867	3.336585
FS-ZKJ-D_{80-85}-216	1.51	7.989	19.631	9.869784
FS-ZKJ-D_{80-85}-217	1.273	7.63	18.548	11.37914
FS-ZKJ-D_{80-85}-218	1.179	14.35	12.639	1.513653
FS-ZKJ-D_{80-85}-219	1.171	15.002	13.399	1.488447
FS-ZKJ-D_{80-85}-220	1.688	15.027	11.509	1.274953
FS-ZKJ-D_{80-85}-221	1.459	7.387	24.433	17.61572
FS-ZKJ-D_{80-85}-222	1.671	7.676	5.592	3.336516
FS-ZKJ-D_{80-85}-223	1.697	14.356	17.936	2.146482
FS-ZKJ-D_{80-85}-224	1.724	11.145	17.66	3.432459

FS-ZKJ-D_{80-85}-225	1.328	9.207	14.916	4.651076
FS-ZKJ-D_{80-85}-226	1.335	13.924	15.463	1.951413
FS-ZKJ-D_{80-85}-227	1.424	15.45	21.325	2.256614
FS-ZKJ-D_{80-85}-228	1.201	9.864	19.885	5.146222
FS-ZKJ-D_{80-85}-229	1.633	10.783	19.75	4.129208
FS-ZKJ-D_{80-85}-230	1.568	9.656	13.55	3.706236
FS-ZKJ-D_{80-85}-231	1.227	8.019	22.486	11.1372
FS-ZKJ-D_{80-85}-232	1.597	14.164	24.858	3.044831
FS-ZKJ-D_{80-85}-233	1.578	11.471	22.82	4.171084
FS-ZKJ-D_{80-85}-234	1.769	12.178	16.463	2.664778
FS-ZKJ-D_{80-85}-235	1.727	8.986	17.679	5.92063
FS-ZKJ-D_{80-85}-236	1.498	9.059	15.987	5.226218
FS-ZKJ-D_{80-85}-237	1.17	9.76	14.068	3.741489
FS-ZKJ-D_{80-85}-238	1.568	14.454	21.188	2.506269
FS-ZKJ-D_{80-85}-239	1.435	8.688	22.238	8.273065
FS-ZKJ-D_{80-85}-240	1.127	12.797	21.749	3.199794
FS-ZKJ-D_{80-85}-241	1.244	13.034	5.806	0.825419
FS-ZKJ-D_{80-85}-242	1.527	8.117	11.862	5.603212
FS-ZKJ-D_{80-85}-243	1.533	15.722	14.512	1.492697
FS-ZKJ-D_{80-85}-244	1.545	10.722	14.192	3.005506
FS-ZKJ-D_{80-85}-245	1.621	13.913	14.001	1.769367
FS-ZKJ-D_{80-85}-246	1.634	14.687	19.351	2.227581
FS-ZKJ-D_{80-85}-247	1.331	7.977	8.31	4.203338
FS-ZKJ-D_{80-85}-248	1.486	7.989	17.776	8.937154
FS-ZKJ-D_{80-85}-249	1.468	12.449	21.778	3.376958
FS-ZKJ-D_{80-85}-250	1.253	14.326	13.214	1.587077
FS-ZKJ-D_{80-85}-251	1.385	7.943	6.776	3.487391
FS-ZKJ-D_{80-85}-252	1.404	9.803	13.192	3.46884
FS-ZKJ-D_{80-85}-253	1.418	13.885	8.533	1.082181
FS-ZKJ-D_{80-85}-254	1.295	11.837	17.832	3.054994
FS-ZKJ-D_{80-85}-255	1.77	7.883	12.543	6.661179
FS-ZKJ-D_{80-85}-256	1.261	12.953	10.243	1.473177
FS-ZKJ-D_{80-85}-257	1.264	9.779	17.226	4.558349

FS-ZKJ-D_{80-85}-258	1.146	7.997	21.12	10.57586
FS-ZKJ-D_{80-85}-259	1.344	13.053	7.133	1.011343
FS-ZKJ-D_{80-85}-260	1.353	15.175	6.351	0.692207
FS-ZKJ-D_{80-85}-261	1.411	12.82	22.26	3.26393
FS-ZKJ-D_{80-85}-262	1.221	8.748	20.505	7.46179
FS-ZKJ-D_{80-85}-263	1.288	15.704	8.841	0.911068
FS-ZKJ-D_{80-85}-264	1.61	11.346	21.508	4.023195
FS-ZKJ-D_{80-85}-265	1.437	7.761	9.333	5.29983
FS-ZKJ-D_{80-85}-266	1.692	11.989	10.112	1.688429
FS-ZKJ-D_{80-85}-267	1.338	10.804	13.316	2.771857
FS-ZKJ-D_{80-85}-268	1.266	13.595	16.697	2.19842
FS-ZKJ-D_{80-85}-269	1.651	9.898	7.157	1.83607
FS-ZKJ-D_{80-85}-270	1.529	11.292	10.066	1.902116
FS-ZKJ-D_{80-85}-271	1.275	13.82	14.796	1.892072
FS-ZKJ-D_{80-85}-272	1.379	14.174	21.405	2.618669
FS-ZKJ-D_{80-85}-273	1.596	9.635	8.77	2.412655
FS-ZKJ-D_{80-85}-274	1.628	13.002	14.57	2.080834
FS-ZKJ-D_{80-85}-275	1.283	14.71	7.683	0.88209
FS-ZKJ-D_{80-85}-276	1.49	10.913	13.352	2.717688
FS-ZKJ-D_{80-85}-277	1.493	11.468	23.427	4.284382
FS-ZKJ-D_{80-85}-278	1.326	7.615	21.126	13.08111
FS-ZKJ-D_{80-85}-279	1.725	14.876	8.71	0.981298
FS-ZKJ-D_{80-85}-280	1.551	8.481	9.867	3.977025
FS-ZKJ-D_{80-85}-281	1.72	8.397	21.887	9.130997
FS-ZKJ-D_{80-85}-282	1.231	9.053	8.13	2.662954
FS-ZKJ-D_{80-85}-283	1.572	11.12	17.416	3.401563
FS-ZKJ-D_{80-85}-284	1.197	8.389	15.242	6.380075
FS-ZKJ-D_{80-85}-285	1.453	14.227	15.518	1.886228
FS-ZKJ-D_{80-85}-286	1.581	13.431	9.586	1.290001
FS-ZKJ-D_{80-85}-287	1.626	14.654	14.356	1.658886
FS-ZKJ-D_{80-85}-288	1.417	9.017	11.82	3.917799
FS-ZKJ-D_{80-85}-289	1.142	12.49	6.053	0.932666
FS-ZKJ-D_{80-85}-290	1.623	10.603	21.862	4.749511

FS-ZKJ-D_{80-85}-291	1.767	8.622	11.796	4.498856
FS-ZKJ-D_{80-85}-292	1.127	12.386	9.113	1.427028
FS-ZKJ-D_{80-85}-293	1.348	15.53	9.723	1.020252
FS-ZKJ-D_{80-85}-294	1.675	7.309	9.251	7.067227
FS-ZKJ-D_{80-85}-295	1.759	14.422	23.756	2.820708
FS-ZKJ-D_{80-85}-296	1.292	15.096	18.671	2.052661
FS-ZKJ-D_{80-85}-297	1.25	12.016	22.254	3.699136
FS-ZKJ-D_{80-85}-298	1.771	14.131	11.895	1.46292
FS-ZKJ-D_{80-85}-299	1.628	11.27	10.233	1.941746
FS-ZKJ-D_{80-85}-300	1.126	12.998	24.959	3.56659
FS-ZKJ-D_{80-85}-301	1.55	10.105	10.186	2.481364
FS-ZKJ-D_{80-85}-302	1.148	12.648	8.198	1.233153
FS-ZKJ-D_{80-85}-303	1.201	13.254	17.156	2.36504
FS-ZKJ-D_{80-85}-304	1.611	10.676	22.127	4.732036
FS-ZKJ-D_{80-85}-305	1.433	12.499	6.084	0.936144
FS-ZKJ-D_{80-85}-306	1.638	8.085	5.533	2.653717
FS-ZKJ-D_{80-85}-307	1.554	7.329	14.495	10.9067
FS-ZKJ-D_{80-85}-308	1.409	15.022	8.698	0.964088
FS-ZKJ-D_{80-85}-309	1.677	14.61	24.417	2.835889
FS-ZKJ-D_{80-85}-310	1.373	12.924	9.012	1.30156
FS-ZKJ-D_{80-85}-311	1.403	15.124	9.556	1.047348
FS-ZKJ-D_{80-85}-312	1.129	11.795	13.985	2.413287
FS-ZKJ-D_{80-85}-313	1.382	7.454	14.06	9.669876
FS-ZKJ-D_{80-85}-314	1.505	11.833	18.1	3.103034
FS-ZKJ-D_{80-85}-315	1.435	14.352	22.884	2.739943
FS-ZKJ-D_{80-85}-316	1.645	13.369	18.078	2.45325
FS-ZKJ-D_{80-85}-317	1.314	10.268	18.215	4.267807
FS-ZKJ-D_{80-85}-318	1.677	12.229	23.245	3.731739
FS-ZKJ-D_{80-85}-319	1.54	12.806	12.514	1.838672
FS-ZKJ-D_{80-85}-320	1.192	11.063	24.725	4.883468
FS-ZKJ-D_{80-85}-321	1.303	12.191	17.76	2.86868
FS-ZKJ-D_{80-85}-322	1.15	13.326	6.232	0.850669
FS-ZKJ-D_{80-85}-323	1.319	7.515	20.809	13.73531

FS-ZKJ-D_{80-85}-324	1.241	9.888	8.438	2.170267
FS-ZKJ-D_{80-85}-325	1.756	14.952	18.799	2.099978
FS-ZKJ-D_{80-85}-326	1.362	14.702	11.288	1.297173
FS-ZKJ-D_{80-85}-327	1.161	9.113	17.598	5.653068
FS-ZKJ-D_{80-85}-328	1.49	15.647	9.728	1.008396
FS-ZKJ-D_{80-85}-329	1.384	11.439	12.093	2.223387
